# THE ROLE OF CASTRATION BEFORE OR AFTER PUBERTY ON SEX DIFFERENCES IN GROWTH AND LONGEVITY IN UM-HET3 MICE

**DOI:** 10.1093/geroni/igad104.1816

**Published:** 2023-12-21

**Authors:** Nisi Jiang, Catherine Cheng, James Nelson

**Affiliations:** UT Health San Antonio, Barshop Institute, San Antonio, Texas, United States; UT Health San Antonio, San Antonio, Texas, United States; UT Health San Antonio, San Antonio, Texas, United States

## Abstract

Sex differences in human aging and longevity are well known. Mortality rates of males are higher throughout life with no definitive mechanisms identified. Previously, we reported that genetically heterogeneous mice have sex differences in longevity like those of humans. Here, we report that prepubertal castration significantly reduced early adulthood mortality and increased median lifespan by 12.6% (n=236/group) to match female levels. It also shifted growth trajectories toward the female pattern, including a reduced rate of body weight gain and a prolonged growth period. Our findings suggest that testicular hormones are critical for these sex differences. We next tested whether a) exposure to gonadal hormones from puberty throughout life is required or b) only hormone exposure during the short period of pubertal development is sufficient to cause these sex differences. We castrated or sham-operated mice (n = 60/group) prepubertally at 20 days, or after puberty at 60 or 240 days. Prepubertal castration prolonged the period of growth as before, with dramatically increased fat mass and reduced lean mass, which reduced the lean/body mass ratio to a level indistinguishable from that of females. Although post-pubertal castrated groups also had reduced lean/body mass ratios, they were significantly higher than prepubertally castrated mice, but were unchanged between 60d and 240d groups. These results suggest that the narrow window of testicular hormone exposure during puberty is necessary for the full expression of sex differences in body composition trajectories. Its effect on lifespan will be presented at the meeting when mice are old enough for assessment.

